# Retraction Note to: Correlations between chromobox homolog 8 and key factors of epithelial–mesenchymal transition in hepatocellular carcinoma

**DOI:** 10.1186/s12935-020-01574-4

**Published:** 2020-10-07

**Authors:** Xiaonian Zhu, Wei Luo, Chunhua Bei, Juan Kong, Shidong Zhang, Yuanyuan Fu, Di Li, Shengkui Tan

**Affiliations:** 1 Department of Epidemiology and Health Statistics, School of Public Health, Guilin Medical University, 109 Huancheng North Road 2, Guilin, 541004 Guangxi People’s Republic of China; 2 Nanchong Central Hospital, The Second Clinical College of North Sichuan Medical College, Nanchong, 637000 Sichuan People’s Republic of China

## Retraction to: Cancer Cell Int (2019) 19:340 10.1186/s12935-019-1063-z

The Editor-in-Chief has retracted this article [1] because three panels in Figure 2D appear to be the same as three panels in Figure 4A in a previously published article by the same author group [2, retracted]. Specifically:

- Invasion/shGFP in Figure 2D [1] appears to be the same as Invasion/shGFP in Figure 4A [2]
- Invasion/shCBX8-1# in Figure 2D [1] appears to be the same as Migration/shGFP in Figure 4A [2]
- Invasion/shCBX8-3# in Figure 2D [1] appears to be the same as Migration/shMAZ-2# in Figure 4A [2]

The data reported in this article are therefore unreliable. None of the authors have responded to any correspondence from the editor or publisher about this retraction.

1. Xiaonian Zhu, Wei Luo, Chunhua Bei, Juan Kong, Shidong Zhang, Yuanyuan Fu, Di Li & Shengkui Tan. Correlations between chromobox homolog 8 and key factors of epithelial–mesenchymal transition in hepatocellular carcinoma. Cancer Cell Int 19, 340 (2019). 10.1186/s12935-019-1063-z
2. Retracted article: Zhu X, Luo W, Liang W, Tang F, Bei C, Ren Y, Qin L, Tan C, Zhang Y, Tan S. Overexpression and clinical significance of MYC-associated zinc finger protein in pancreatic carcinoma. Oncotargets and Therapy 2016(9), 7493–7501 (2016)

